# Characterization, Quantification and Quality Assessment of Avocado (*Persea americana* Mill.) Oils

**DOI:** 10.3390/molecules25061453

**Published:** 2020-03-24

**Authors:** Mei Wang, Ping Yu, Amar G. Chittiboyina, Dilu Chen, Jianping Zhao, Bharathi Avula, Yan-Hong Wang, Ikhlas A. Khan

**Affiliations:** 1 National Center for Natural Products Research, School of Pharmacy, University of Mississippi, University, MS 38677, USA; meiwang@olemiss.edu (M.W.); amar@olemiss.edu (A.G.C.); jianping@olemiss.edu (J.Z.); bavula@olemiss.edu (B.A.); wangyh@olemiss.edu (Y.-H.W.); 2 State Key Laboratory of Food Science and Technology, Nanchang University, Nanchang 330031, China; cpu_yuping@126.com; 3 Jiangxi Province Key Laboratory of Edible and Medicinal Resources Exploitation, Nanchang University, Nanchang 330031, China; 4 School of Resource and Environmental and Chemical Engineering, Nanchang University, Nanchang 330031, China; 5 School of Pharmacy, Hunan University of Chinese Medicine, Changsha 410208, China; cdl918@126.com; 6 Division of Pharmacognosy, Department of BioMolecular Sciences, School of Pharmacy, University of Mississippi, University, MS 38677, USA

**Keywords:** *Persea americana* Mill., avocado oil, triglyceride, fatty acid, UHPLC/ESI-MS, GC/MS, quality evaluation

## Abstract

Avocado oil is prized for its high nutritional value due to the substantial amounts of triglycerides (TGs) and unsaturated fatty acids (FAs) present. While avocado oil is traditionally extracted from mature fruit flesh, alternative sources such as avocado seed oil have recently increased in popularity. Unfortunately, sufficient evidence is not available to support the claimed health benefit and safe use of such oils. To address potential quality issues and identify possible adulteration, authenticated avocado oils extracted from the fruit peel, pulp and seed by supercritical fluid extraction (SFE), as well as commercial avocado pulp and seed oils sold in US market were analyzed for TGs and FAs in the present study. Characterization and quantification of TGs were conducted using UHPLC/ESI-MS. Thirteen TGs containing saturated and unsaturated fatty acids in avocado oils were unambiguously identified. Compared to traditional analytical methods, which are based only on the relative areas of chromatographic peaks neglecting the differences in the relative response of individual TG, our method improved the quantification of TGs by using the reference standards whenever possible or the reference standards with the same equivalent carbon number (ECN). To verify the precision and accuracy of the UHPLC/ESI-MS method, the hydrolysis and transesterification products of avocado oil were analyzed for fatty acid methyl esters using a GC/MS method. The concentrations of individual FA were calculated, and the results agreed with the UHPLC/ESI-MS method. Although chemical profiles of avocado oils from pulp and peel are very similar, a significant difference was observed for the seed oil. Principal component analysis (PCA) based on TG and FA compositional data allowed correct identification of individual avocado oil and detection of possible adulteration.

## 1. Introduction

Avocado (*Persea americana* Mill.) is a member of the Lauraceae family. Although avocado trees are native to Central America, they are also widely distributed in tropical and subtropical countries. Anatomically, the avocado fruit can be distinguished into three regions - the innermost seed that constitutes 20% of the fruit, the pulp covering the major portion (65%) and the outermost peel (15%) [1,2]. Popularly known as “vegetable butter” or “butter pear”, the fruit contains a substantial amount of triglycerides (TGs) along with a high content of unsaturated fatty acids. It is also rich in many other bioactive phytochemicals such as carotenoids, tocopherols, phytosterols, aliphatic alcohols and hydrocarbons [3,4].

Unlike oil extracted from other fruits, the oil from avocado fruit is often extracted from the mature fruit flesh [4], and its lipid content has been reported as the highest among all known fruit and vegetable varieties [5,6,7]. Avocado oil has a multitude of applications such as a culinary oil and as an ingredient in healthcare products, cosmetics, pharmaceuticals and nutraceuticals. The consumption of avocado oil has become popular owing to its high nutritional value and potential benefit to human health, including the management of hypercholesterolemia [8,9], hypertension [10], diabetes and fatty liver disease [11]. The oil can also reduce cardio-metabolic risk [12] and possesses anti-cancer and antimicrobial properties [13,14]. Over the last decade, the production of avocado oil worldwide has grown steadily and currently accounts for about 4.4 million tons of fresh fruit [15,16].

TGs are the most important nutritive group of compounds in avocado oil and represent a significant amount (~90%) of the entire oil composition. Chemically, TGs are complex hydrophobic molecular species formed by the esterification of three fatty acids (FAs) with a glycerol backbone under enzymatic catalysis. The complexity of TGs is due to a large number of possible FA combinations attached to the glycerol skeleton, which can differ in the number of acyl carbon atoms (CNs), the degree of unsaturation, and the position and configuration (cis/trans) of the double bonds (DBs) in each FA. Furthermore, the TG molecule demonstrates optical activity (enantiomers) when the two primary hydroxyl groups are esterified with different FAs, and the stereo-specific distribution (regioisomers) can vary when stereo-chemical positions (sn-1, 2 or 3) on the glycerol skeleton are attached by various combinations of FAs. Several analytical techniques have been employed for the qualitative and quantitative determination of TGs in edible oils, ranging from spectroscopy methods such as infrared spectroscopy [17,18] and nuclear magnetic resonance [19] to chromatographic techniques including gas chromatography (GC), liquid chromatography (LC) [20] and supercritical fluid chromatography (SFC) coupled with mass spectrometry/tandem mass spectrometry [21]. Non-aqueous reversed-phase liquid chromatography coupled with positive-ion atmospheric pressure chemical ionization (APCI) mass spectrometry has become increasingly popular and currently is the most widely used separation technique for TGs analysis. By using this technique, the separation of TGs is governed by the equivalent carbon number (ECN) defined as ECN = CN – 2DB. Separations of TGs within the same ECN group [22,23], cis/trans isomers and isomers with different positional DB have been reported [24]. In contrast, GC is the most commonly used method for the analysis of FAs, but it requires transesterification to convert TGs to its corresponding fatty acid methyl esters (FAMEs). Although high-temperature GC for the direct determination of intact TGs has been reported [25], samples subjected to this technique must be thermally stable and resistant to isomerization.

In recent years, the popularity of avocado oil in the US market has been promoted with oils extracted from alternative sources such as avocado seed. Some manufacturers and consumers have considered avocado seed oil as a source of fatty acids, carbohydrates, dietary fiber and a broad range of phytochemicals. Unfortunately, there is no sufficient evidence to support the claimed health benefits and safe use of such oils. In addition, vegetable oils are among the top 25 ingredients that are most susceptible to adulteration and represent 24% of reported fraud cases [18]. Thus, avocado oil could be a target for fraudulent practices such as adulteration with low-cost oils. Therefore, the development of accurate and reproducible methods for TG and FA analysis in avocado oil is needed for characterization and quality control of this valuable commodity.

As part of an ongoing research program on the authentication, safety and biological evaluation of phytochemicals and dietary supplements, an in-depth chemical investigation of avocado oil was performed. The current study aimed to establish the comprehensive profile of TGs in oils extracted from avocado peel, pulp and seed. A UHPLC/ESI-MS method was developed for the identification and quantification of 13 TGs present in authenticated and commercial avocado oils. Furthermore, the hydrolysis and transesterification products of avocado oils were analyzed for FAMEs using a GC/MS method. To verify the precision and accuracy of the developed methods, the results from GC/MS and UHPLC/ESI-MS were compared. Finally, the TG and FA compositional data, along with chemometric analysis, was used for quality evaluation and identification of possible adulteration in commercial oils.

## 2. Results and Discussion

### 2.1. Supercritical Fluid Extraction of Avocado Oil

Generally, avocado oil is extracted from avocado pulp by centrifugation, cold pressing or solvent extraction [26]. These extraction methods are time-consuming and economically unfavorable. Supercritical fluid extraction (SFE), on the other hand, is an environmentally friendly and cost-effective extraction method with a multitude of applications in the food, pharmaceutical and fine chemical industries. In the present study, SFE was used for the extraction of oils from avocado peel, pulp and seed. The yields of the oils along with their physicochemical properties determined by the standard method of the American Oil Chemists’ Society [27] were compared with the oils extracted with the AOAC method [28]. The results are given in Table 1. The color of the oils extracted from the SFE method was much lighter than that of the solvent extraction, indicating a smaller amount of chlorophyll being extracted. The TG and FA profiles for these two extraction methods were similar as determined by UHPLC/MS and GC/MS analysis. Therefore, the oils extracted by SFE were used for further studies.

### 2.2. TGs Profile of Avocado Oils

#### 2.2.1. Method Development and Optimization

**Figure 1:**
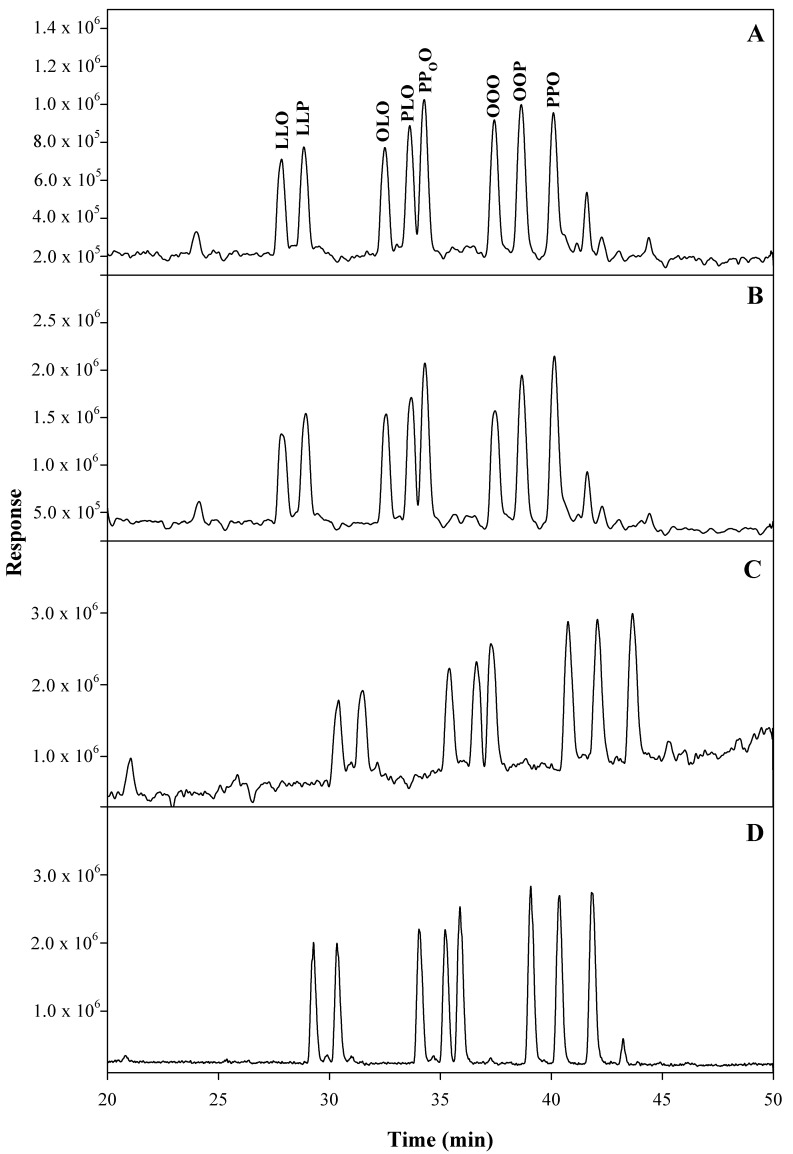
Total ion chromatograms of (**A**) APCI(+), eluent: acetonitrile/isopropanol; (**B**) ESI(+), eluent: acetonitrile/isopropanol; (**C**) ESI(+), eluent: acetonitrile with 0.05% formic acid/isopropanol with 0.05% formic acid and 5 mM ammonium formate; (**D**) ESI(+), eluent: acetonitrile with 0.1% water and 0.05% formic acid/isopropanol with 0.05% formic acid and 5 mM ammonium formate.

**Figure 2:**
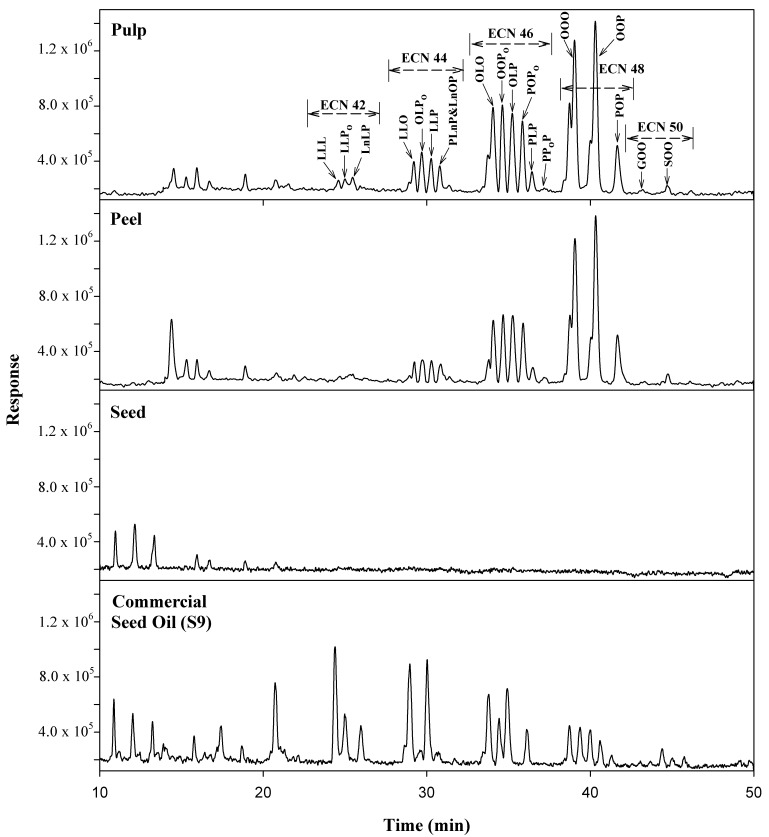
Total ion chromatograms of authenticated avocado oils extracted from pulp, peel, seed and commercial avocado seed oil. ECNs: the equivalent carbon numbers.

Avocado oils are characterized by a high content of TGs. The separation and unambiguous identification of structurally similar TGs in avocado oil pose great analytical challenges. In this study, 12 commercially available TG reference standards were purchase (Table 2) and used for method development and quantification. The method was optimized regarding the chromatographic column, eluent and gradient program. Different reversed-phase columns including several Agilent ZORBAX columns such as Eclipse Plus C_18_, SB-C_18_, XDB-C_18_, SB-C_8_ (with the same dimensions of 2.1 × 100 mm × 1.8 µm), and ACQUITY UPLC BEH C_18_ (Waters, 2.1 × 100 mm × 1.7 µm) as a standalone column or in a combination were investigated. Finally, three ACQUITY UPLC BEH C_18_ connected in series were used to provide the best separation of the targeted TGs. For the evaluation of eluents, unlike non-aqueous eluents used by the majority of TGs analyses [21,29,30,31], eluent consisting of acetonitrile with 0.1% water (v/v) and isopropanol with 5 mM ammonium formate was used, and the chromatographic peak shapes were greatly improved. The chromatograms showing the method optimization using a mixed solution containing LLO, LLP, OLO, PLO, PPoO, OOO, OOP and PPO standards are illustrated in Figure 1. The chromatograms of authenticated oils extracted from avocado peel, pulp and seed, as well as one of the commercial products claimed as avocado seed oil, are shown in Figure 2. It is worth noting, as the ECNs increased from 44 to 50, the retention times for the corresponding TGs also increased (Figure 2). Although similar TG profiles of avocado peel and pulp oils were observed, a significant difference was present in avocado seed oil. The profile for the commercial avocado seed oil appeared to be dramatically different with any of the other avocado oils, suggesting that this particular oil was possibly adulterated with other oils.

#### 2.2.2. Identification of TGs

**Figure 3:**
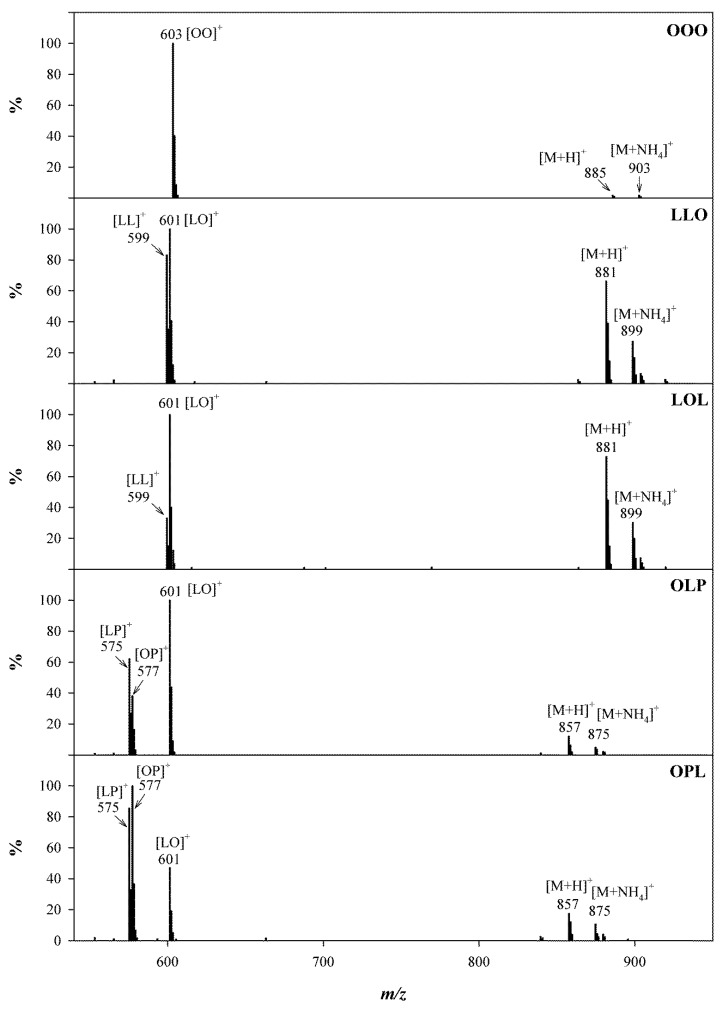
Positive ion ESI spectra of TGs containing different acyls on the glycerol backbone. OOO, LLO and LOL, and OLP and OPL were used as representative examples for single-acid type (R_1_R_1_R_1_), mixed-acid type (R_1_R_2_R_1_ or R_1_R_1_R_2_) and mixed-acid type (R_1_R_2_R_3_), respectively.

**Figure 4:**
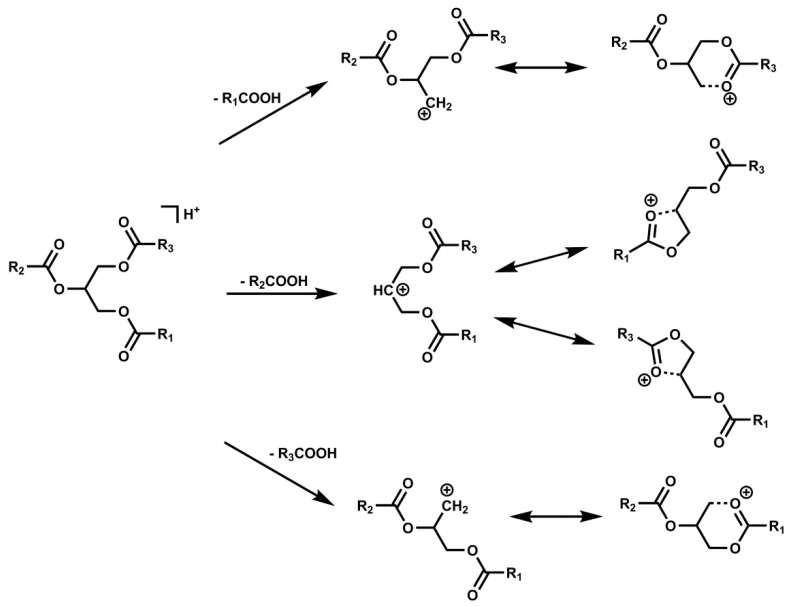
Schematic representation of fatty acids elimination from sn-1, sn-2 and sn-3 positions by ESI(+) MS.

Unambiguous identification of complex TGs in avocado oil is desirable for the accurate quantification of TGs. Many HPLC detection techniques including refractive index [32], UV-Vis and evaporative light-scattering (ELSD) [21], have been applied for the qualitative analysis of TGs in plant oils. Although each of these detection methods has its own advantages, it may not be possible to confidently identify TGs with complete or even partial chromatographic resolution in complex plant oils that contain numerous species with the same ECNs. In our study, both APCI and ESI with positive/negative ion modes were evaluated, and ESI(+) provided better sensitivity for all the TGs with the optimized solvent system (Figure 1D). The ESI(+) mass spectra and notation of fragment ions for representative TGs are illustrated in Figure 3. As described before, TGs in plant oils usually exist as a mixture of positional isomers differing by the acyl attachment, sn-1, sn-2 and sn-3. The [M + H]^+^ and [M + NH_4_]^+^ ions were detected in all TGs with relatively low abundances compared to the corresponding [M + H-RCOOH]^+^ ions. Except for these two ions, single-acid type (R_1_R_1_R_1_) provided only one ion such as [OO]^+^ in OOO. Mixed-acid type (R_1_R_2_R_1_ or R_1_R_1_R_2_) always provided two different ions, such as [LL]^+^ and [LO]^+^ for both LLO and LOL types TGs. Conversely, three different ions were observed for mix-acid type (R_1_R_2_R_3_), such as [LP]^+^, [LO]^+^ and [OP]^+^ for OLP and OPL. All the examples are shown in Figure 3. Although the theoretical ion abundance ratios of [LO]^+^/[LL]^+^ should be 2:1 for both LLO and LOL, different values (1.2 for LLO and 3.0 for LOL, respectively) were observed. A similar observation was achieved for OLP and OPL. The theoretical ion abundance ratios of [LP]^+^/[OP]^+^/[LO]^+^ should be 1:1:1 for both OLP and OPL, whereas the measured values were 0.62:0.38:1 for OLP, and 1.8:2.1:1 for OPL. This observation indicated that the loss of FA from the equivalent sn-1 and sn-3 positions is preferred rather than the middle position sn-2. Figure 4 proposed the possible mechanism for the cleavage of fatty acids from TGs at different positions. Based on observed fragments, the formation of a 5-member ring as a result of losing FA group in the sn-2 position is less favorable than the formation of a more stable 6-member ring in position sn-1 or sn-3 [33]. The relative abundances of ions formed from the loss of FAs in different positions in the MS afford the confident identification of the acyl position on the glycerol backbone.

**Figure 5:**
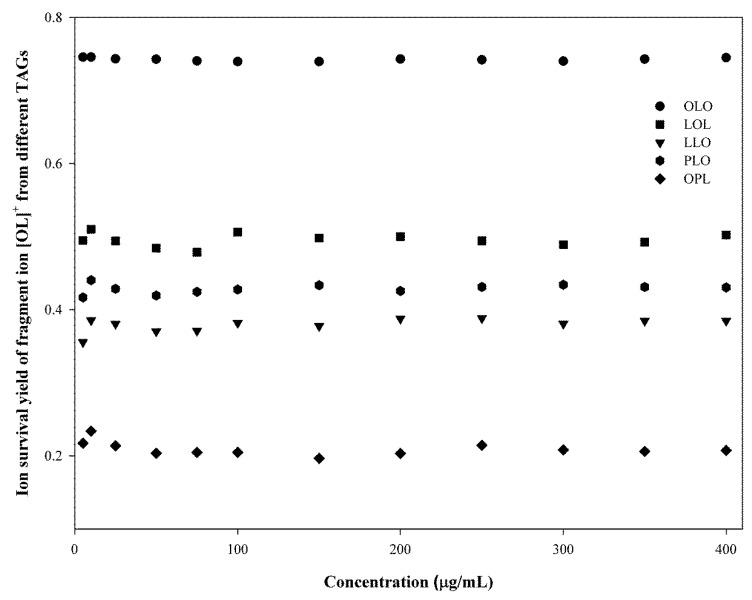
Ion survival yields (ISYs) for the fragment ion [OL]^+^ from representative TGs over the concentration range.

An alternative approach for the identification of TGs in avocado oils is to measure the ion survival yield (ISY). As demonstrated in Figure 3, various types of ions were formed by in-source collision-induced dissociation (IS-CID). The production of information-rich fragments by IS-CID can materially aid in the identification of components, elucidation of structures, and distinction between isomers and chemically similar components in complex mixtures [34]. Ion distribution in IS-CID has been commonly measured by ISY defined as ISY=IaIa+∑Ib where $I_{a}$ represents the measured intensity of the monitored ion and $I_{b}s$ are the intensities of the additional ions formed in the source. Accurate qualitative and quantitative analysis requires that the ISYs of reference standards should be independent of the concentrations of standards, and the ISYs should be equivalent to the standards and analyzed samples. The ISYs for the representative TG standards were measured over the range of 5–400 μg/mL (Figure 5). The ISYs for the fragment ion of [LO]+ after the loss of one FA from different positions (sn-1, sn-2 or sn-3) were calculated from the representative TGs. For example, LLO and LOL both displayed a pseudo-molecular ion [M + H]^+^ at 881 with a fragment ion [LO]^+^ at 601. However, the ISY for [LO]^+^ generated from LLO was 0.38 (±0.01), whereas that from LOL was 0.50 (±0.01). Similarly, ISYs for [LO]^+^ from PLO and OPL were 0.43 (±0.01) and 0.21 (±0.01), respectively, suggesting that the measured ISYs can be used for the identification and characterization of TGs in complex samples [35].

#### 2.2.3. Quantification of TGs

**Figure 6:**
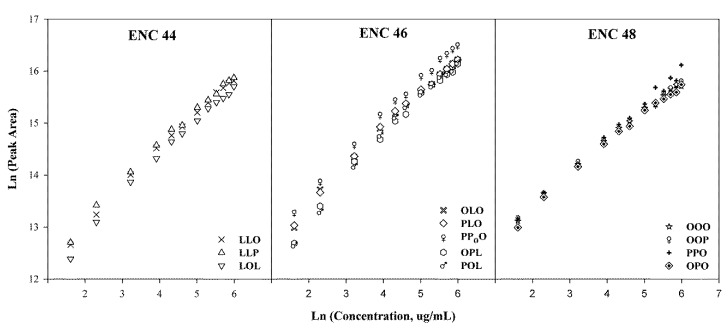
Calibration curves (grouped by ECN) for 12 commercially available reference standards.

A prolonged challenge in the accurate quantification of TGs is the lack of commercially available reference standards. Natural TGs are complex, and commercial standards are available only for a limited number, mostly single-acid (R_1_R_1_R_1_) type. Thus, quantification based on the calibration curve from each individual TG reference standard is practically impossible. Previously, the quantification of TGs was based on the relative peak areas neglecting the differences in the relative response for each individual TG. Later on, a more sophisticated approach using response factors (RFs) was reported by Holcapek, et al. [31], in which the calibration curves of single-acid TGs were measured, and the RFs of mixed-acid TGs expressed relative to the most common OOO were calculated. In our study, 12 commercially available TG reference standards were used for the TGs quantification. Calibration curves for the 12 standards were realized by plotting the logarithms of the sum peak areas of all ions from IS-CID for each TG versus logarithms of analyte concentrations as shown in Figure 6. The results exhibited good linearity (R^2^ > 0.99) over the concentration range of 1–400 µg/mL, and the limits of quantification were 1 µg/mL for all the analytes. When the total content of TGs was calculated, the recovery values were all in the range of 95%–107% (RSD < 7%), and the RSD values of precision, including intra-day and inter-day, were determined to be < 7%. Interestingly, the calibration curves for TGs within the same ECN group were nearly overlapped as shown in Figure 6, suggesting that the quantification method could select one single standard from each ECN group, and simultaneously determine multiple compounds in the same ECN group when reference standards are commercially unavailable. This single standard method could significantly lower the cost and time of the experiment [36].

The authenticated avocado peel, pulp and seed oils, sesame oil and soybean oil which have been reported as potential adulterants [17], along with 19 commercial avocado pulp or seed oils (Table S1) were quantified for the 13 characteristic TGs identified in avocado oils. The quantification results are given in Table 3. The quantification of all the TGs in the seed oil was below the detection limit of the current method. In addition, the yield from avocado seed oil was less than 2%, demonstrating that any commercial avocado seed oil sold in US market would be questionable due to the poor economic viability. Avocado peel and pulp oil showed very similar chemical profiles. The total compositions of the 13 TGs were 67.4% in the peel oil and 86.3% in the pulp oil, respectively. OOO and OOP are the most prominent TGs, accounting for ~25% of total TG contents in both peel and pulp oils, followed by OLO (~18%) and OOPo (~7%). On the other hand, soybean and sesame oils showed significant differences (Table 3). OLO is the most abundant compound in both sesame oil (~42%) and soybean oil (~29%), whereas OOO and OOP are relatively low, accounting for 10% and 2.5% in sesame oil and 6% and 3% in soybean oil, respectively. Other TGs, such as LLL have been detected in both sesame and soybean oils but were not identified in avocado oil. Among the analyzed 19 (S1–S19) commercial avocado pulp and seed oils purchased in the US market, S10 and S19, claimed as avocado seed oils, demonstrated very similar chemical profiles as avocado pulp oil. S4 and S9, claimed to be avocado seed oil, along with S6, S7 and S16 (plant parts were not specified), showed significant differences to avocado pulp oil, but exhibited similar profiles to soybean oil. These samples could possibly be adulterated with soybean oil. S17 was claimed as avocado pulp oil but demonstrated a profile close to a combination of avocado and sesame oils.

### 2.3. FAs Profile of Avocado Oils

Except for the most abundant compounds of TG in avocado oil, the derived FA composition can also be exploited as a peculiar fingerprint indicative of the oil’s quality and authenticity [37]. FAs are a group of very complex compounds, including monounsaturated FAs, polyunsaturated FAs and saturated FAs. In this study, the selected HP-88 with (88% cyanopropy)aryl-polysiloxane stationary phase GC capillary column is a high-polarity column designed for the separation of FAMEs including those positional cis/trans isomers. All the authenticated and commercial oils were transesterified and analyzed by GC/MS. The FA profile comprised a total of seven FAs in avocado oils, viz. palmitic acid, palmitoleic acid, stearic acid, oleic acid, vaccenic acid, linoleic acid and linolenic acid. Oleic acid was the major FA (~60%), followed by palmitic acid (~15%), linoleic acid (~10%), palmitoleic acid (~7%) and vaccenic acid (~6%). This agreed with the individual FA moiety identified in the TGs by UHPLC/ESI-MS. The quantification results are summarized in Table 4. Again, the FA profiles of soybean and sesame oils showed significant differences from the avocado oils. Oleic acid was the most abundant FA (~44%) in sesame oil, followed by linoleic acid (~40%), palmitic acid (~9%) and stearic acid (~5%). In soybean oil, linoleic acid (~57%) was the major FA, followed by oleic acid (~20%), palmitoleic acid (~11%) and linolenic acid (~7%). Interestingly, avocado oils contain much less stearic acid (~0.3%) compared to soybean oil (~4%) and sesame oil (~5%). The FA compositions of the commercial samples were evaluated. Similar to the TGs analysis, S4, S6, S9, S16 and S17 contained a relatively high concentration of stearic acid (3.0%–4.8%), like sesame and soybean oils, and the FA profiles were significantly different with the authenticated avocado oil. Therefore, these five samples are likely adulterated.

To verify the precision and accuracy of the developed UHPLC/ESI-MS method, the composition data of TG and FA obtained from UHPLC and GC were compared to the authenticated avocado peel and pulp oils. The concentration of individual FA was calculated for all the identified TGs. Oleic acid and vaccenic acid are structurally similar and only differed in double bond positions and these two FAs could not be separated with UHPLC/MS. Thus, they were combined when compared with the TG data. The comparison of TG and FA data is summarized in Table 5. The measurement of TGs by the UHPLC/MS method might be affected by several factors, such as: i) one FA may be distributed among many different combinations in TGs, resulting in a TG concentration below the detection limit [31]; ii) the trace amount of mono/diacylglycerols were not calculated in the TGs method; and iii) the coelution of TGs may complicate the identification of trace FAs. All these factors may result in making the total compositions of TGs slightly lower than FAs. Taking all the factors into account, the data from LC and GC methods were consistent and within an acceptable experimental error range.

### 2.4. Identification of Adulteration Using Chemometric Method

**Figure 7:**
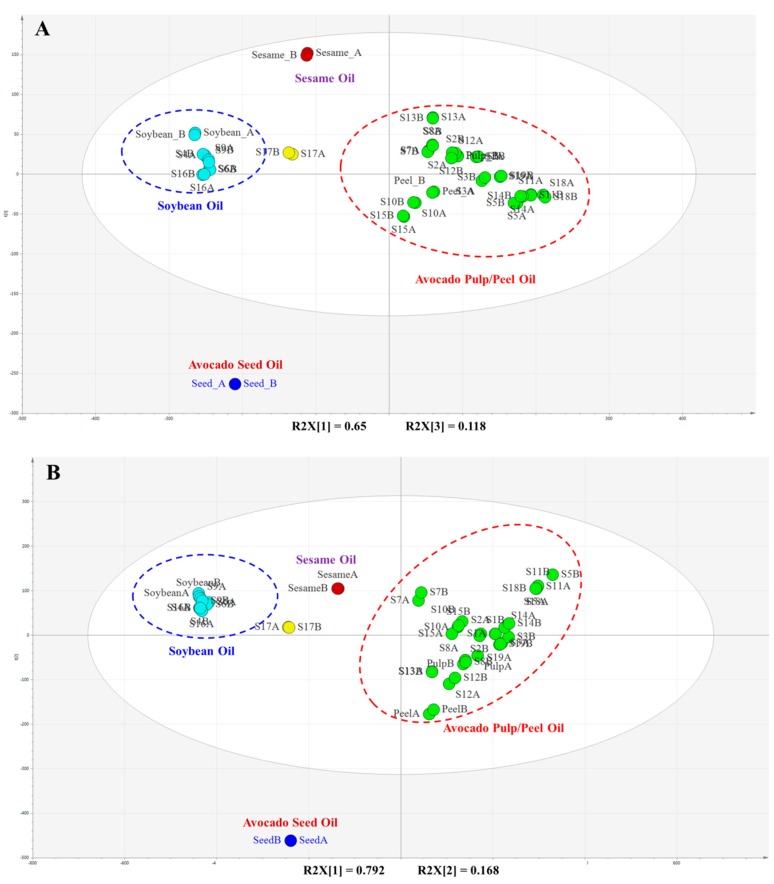
PCA score plots of authenticated and commercial avocado oils, sesame oil and soybean oil. (**A**) UHPLC/MS TG data and (**B**) GC/MS FA data. Each sample was prepared in duplicate.

To further identify possible adulteration, both UHPLC/MS and GC/MS data were subjected to multivariate data reduction chemometric analysis consisting of the principal component analysis (PCA). The PCA score plots are shown in Figure 7A,B for the UHPLC/MS and GC/MS data, respectively. Distinctive groups were observed in both techniques. In each plot, the avocado peel and pulp oils were grouped together, whereas the avocado seed oil was much further away from the peel and pulp oil (Figure 7A,B). None of the samples claimed as avocado seed oils (S4, S9, S10 and S19) were clustered with the seed oil. Instead, S10 and S19 were grouped with pulp and peel oils, and S4 and S9 were grouped closely with soybean oil. S6 and S16 were labeled as avocado oil, but PCA indicated soybean oil as a possible adulterant. S17 might be a mixture of avocado oil with soybean or sesame oil. The adulteration identified by PCA further confirmed the results from both UHPLC/MS and GC/MS analyses.

## 3. Materials and Methods

### 3.1. Chemicals and Materials

*n*-Hexane (GC grade), 2-propanol (Optima LC/MS grade) and ammonium formate (HPLC grade) were purchased from Sigma-Aldrich (St. Louis, MO, USA). Formic acid obtained from Honeywell (Waltham, MA, USA) was of HPLC grade. Water was obtained from a Milli-Q system (Millipore, Burlington, MA, USA). The reference standards of soybean oil and sesame oil (Analytical grade) were also obtained from Sigma-Aldrich.

TG standards: OOO was purchased from Nu-Chek-Prep, Inc. (Elysian, MN, USA). LLO, LLP, OLO, PLO, OOP, PPO, PPO_O_, LOL, OPL, OPO and POL (Table 2) were purchased from Larodan (Monroe, MI, USA). The purities of all the TGs standards were >99.0% as per the label and further confirmed by peak area normalization with LC/MS analysis.

FAMEs standards: the methyl esters of undecanoic acid, palmitic acid, palmitoleic acid, steric acid, oleic acid, vaccenic acid, linoleic acid and linolenic acid were obtained from Nu-Chek-Prep, Inc. The purities of all the FAMEs standards were >99.0% by the label and further confirmed by peak area normalization with GC/MS analysis. The FAMEs mixture (C_4_–C_24_) consisting of 36 compounds with the positional DB and cis/trans configuration isomers was purchased from Sigma-Aldrich and used for further compound identification.

Mature fresh fruits of avocado (*P. americana*) were purchased from different local grocery stores located in Oxford, MS, USA. All the fruits were selected manually with good morphological integrity. The authenticity of the avocado fruits was confirmed by Dr. John Sabestian, a taxonomist at the National Center of Natural Products Research (NCNPR), University of Mississippi. After washing and drying at room temperature, the peel, pulp and seed were separated and cut into small pieces. The isolated parts were freeze-dried for 24 h until constant weights were obtained. The dehydrated samples were then kept in sealed containers and stored at –20 °C to avoid any possible degradation and content loss.

Nineteen avocado oil products claimed to contain avocado pulp or seed oil were purchased from different grocery stores in the US or via various online commercial vendors (Table S1). Each of these 19 commercial samples, along with the authenticated avocado fruits, was assigned a unique identification code, and representative voucher samples were deposited in the Botanical Repository of NCNPR at the University of Mississippi.

### 3.2. Sample Preparation

Two extraction methods, viz. solvent extraction and SFE, were performed and evaluated. The solvent extraction was conducted by following the method described in AOAC 920.39 [28]. Five grams of each avocado peel, pulp or seed (dried powder) was extracted using a Soxhlet apparatus with 200 mL n-hexane at 70 °C for 4 h, and the procedure was repeated once. For the SFE, the same amount of each sample was loaded into the extraction vessel and mixed with glass beads. The extraction parameters, such as CO_2_ flow rate, extraction time, pressure and temperature as well as co-solvent were optimized to obtain the highest oil yields possible. Finally, the CO_2_ flow rate of 10 mL/min, 250 bar, 50 °C and 40 min were adopted. For both extraction methods, the oil yields were calculated as the percentage of oil obtained based on the weight of the sample used. The solvent extraction was used as a reference method for the comparison of oil yields obtained from SFE method.

### 3.3. Determination of TGs Using UHPLC/ESI-MS

Stock solutions of LLO, LLP, OLO, PLO, OOP, PPO, PPoO, OOO, LOL, OPL, OPO and POL at the concentration of 5 mg/mL were prepared in *n*-hexane:isopropanol (1:1, *v*/*v*). These solutions were diluted with the same solvent mixture yielding 12 calibration working solutions between 1–400 µg/mL. All the oil samples were prepared in 1 mg/mL and 500 µg/mL prior to chromatographic analysis.

The chromatographic system consisted of an Agilent 1290 Infinity series UHPLC with a diode array detector, binary pump, auto-liquid sampler and thermostated column compartment (Santa Clara, CA, USA). The UHPLC conditions were: three ACQUITY UPLC BEH C_18_ columns (3.0 ×100 mm, 1.7 µm, Waters, Milford, MA, USA) were connected in series with the column temperature set to 30 °C. The eluents consisted of acetonitrile with 0.1% water (A), isopropanol with 5 mM ammonium formate (B), and both contained 0.05% formic acid. The gradient elution started at 20% B, programmed to 50% B in 45 min, and then 60% B in 35min. The flow rate was 0.3 mL/min, and 1 µL of each solution was injected in duplicate for the LC analysis.

The UHPLC instrument was coupled to an Agilent 6120 quadrupole mass spectrometer with a dual ESI and APCI interface. Both ESI and APCI in positive and negative modes were evaluated, and ESI(+) in full scan mode from 300–1000 amu was selected for the analysis of TGs. The fragmentor voltage was optimized to 140 V. The drying gas flow was 12 L/min and the nebulizer pressure was 35 psi. The drying gas temperature and vaporizer temperature were set to 325 °C and 250 °C, respectively. The capillary voltage was 4000 V and the corona current was 4.0 µA.

### 3.4. Determination of FAs Using GC/MS

Stock solutions of palmitic acid, palmitoleric acid, stearic acid, oleic acid, vaccenic acid, linoleic acid and linolenic acid in the form of methyl ester were prepared in *n*-hexane to make the 10 mg/mL stock solutions. These solutions were diluted with the same solvent yielding 12 calibration working solutions between 5–1000 µg/mL. Undecanoic acid methyl ester was used as the internal standard and added to each calibration solution at a fixed concentration of 200 µg/mL.

A modified procedure based on the AOAC 996.06 method [28] and the method proposed by Ai, et al. [38] for the hydrolytic reaction and transesterification of TGs was employed. In brief, 70 µL of each oil sample was accurately weighed (57.2–67.7 mg) and mixed with 2 mL glyceryl triundecanoate (2.5 mg/mL) and 200 µL 2N potassium hydroxide, both in methanol. The resulting cloudy solution was vortexed for 2 min, and then sonicated for 45 min at 60 °C in a water bath until the solution became clear. Then, 2 mL *n*-hexane was added to the solution. After sonication for 30 min and centrifugation, the supernatants were taken and diluted (2, 4 and 10 times) to obtain three different concentrations for GC/MS analysis.

The FAMEs analysis was performed on an Agilent 7890B gas chromatography (Santa Clara, CA, USA) coupled with an Agilent 5977A mass spectrometer. An Agilent J&W HP-88 column (60 m × 0.25 mm × 0.20 µm) was used. Helium was used as the carrier gas at a constant flow rate of 1.2 mL/min. The oven temperature was first set at a 60 °C hold for 1 min, and the temperature was subsequently increased to 145 °C at a rate of 10 °C/min, then to 190 °C at a rate of 1 °C/min, and finally to 240 °C at a rate of 5 °C/min. The inlet temperature was 260 °C. The split ratio was 100:1 with 1 µL injection. Full scan data was acquired in the mass range of *m/z* 30–500 amu and the EI voltage at 70 V. The temperature of the transfer line was 260 °C. The temperatures of ion source and quadrupole were set to 230 °C and 150 °C, respectively.

### 3.5. Statistical Analysis

The raw data for both UHPLC/MS and GC/MS were pre-processed using MassHunter Profinder (version 8.0, Agilent Technologies, Santa Clara, CA, USA) for finding features. The extracted features were exported as a cef file and then imported to Mass Profiler Professional software package (version B.12.05, Agilent Technologies) and SIMCA-P software (Version 12.0, Umetrics, Umeå, Sweden) where the features were further aligned, normalized and statistically evaluated.

## 4. Conclusions

The current study endeavors to establish the comprehensive profiles and quality standards of avocado oil. The “solvent-free” SFE method used for avocado oil extraction is highly recommended in the food industry. Two independent and complementary analytical methods (LC and GC) were used to investigate different classes of compounds (TG and FA) in avocado oils. Characterization and quantitative analysis of 13 TGs in oils extracted from different parts of avocado fruit, viz. peel, pulp and seed, were conducted using UHPLC/ESI-MS. The complex TGs can be conclusively identified using the MS detection with the correct attribution of the acyl in the sn-2 position, as well as the calculation of ISYs for ions formed in the IS-CID. The efficiency and accuracy of the quantification were improved by the selection of one single reference standard from the same ECN group when reference standards are commercially unavailable. The FA compositions yielded from GC/MS method were in good agreement with UHPLC/MS. Although chemical profiles of avocado pulp and peel were very similar, a significant difference was observed for the avocado seed. It is concluded that the combination of TG and FA analysis using UHPLC/ESI-MS and GC/MS, as well as multivariate statistical analysis may provide comprehensive information for the characterization, standardization and authentication of avocado oils. The reported techniques might be useful for assessing the quality of other plant oils.

## Figures and Tables

**Table 1:** Extraction conditions, oil yields and physicochemical properties (mean ± SD, *n* = 3).

Extraction Method		SFE			Solvent		
Extraction Conditions	Solvent	CO_2_			*n*-Hexane		
	Time (min)	40			480		
	Temperature (°C)	50			70		
	Pressure (bar)	250			atmospheric		
Sample		Peel	Pulp	Seed *^,b^	Peel	Pulp	Seed *^,b^
Oil Yield *^,a^ (%)		16.89 ± 0.56	56.10 ± 0.66	1.61 ± 0.31	17.32 ± 0.30	58.35 ± 0.27	1.66 ± 0.61
Physicochemical Properties	Acid Value (g/oleic acid 100 g)	1.60 ± 0.13	0.94 ± 0.03	N/A	0.69 ± 0.03	0.38 ± 0.00	N/A
	Saponification Value (mg KOH/g)	186.90 ± 0.74	193.71 ± 1.05	N/A	182.15 ± 0.11	186.15 ± 0.88	N/A
	Iodine Value (g I_2_/100 g)	80.62 ± 0.38	90.76 ± 0.46	N/A	72.38 ± 0.41	88.40 ± 0.91	N/A
	Peroxide Value	2.36 ± 0.13	1.18 ± 0.02	N/A	3.53 ± 0.09	2.41 ± 0.13	N/A

*^,a^: Oil yields were calculated based on the dry weight. *^,b^: Not enough quantity to perform the measurements.

**Table 2:** Information of triglyceride reference standards.

No.	Compound	Abbr.	ECN	MW	Formula	CAS #
1	1,3-linolein-2-olein	LOL	44	881.40	C_57_H_100_O_6_	2190-22-9
2	1,2-linolein-3-olein	LLO	44	881.40	C_57_H_100_O_6_	2190-21-8
3	1,2- linolein-3-palmitin	LLP	44	855.36	C_55_H_98_O_6_	2190-15-0
4	1,3-olein-2-linolein	OLO	46	883.42	C_57_H_102_O_6_	2190-19-4
5	1-olein-2-palmitin-3-linolein	OPL	46	857.38	C_55_H_100_O_6_	2534-97-6
6	1-palmitin-2-linolein-3-olein	PLO	46	857.38	C_55_H_100_O_6_	2680-59-3
7	1-palmitin-2-palmitolein-3-olein	PPoO	46	831.34	C_53_H_98_O_6_	81637-60-7
8	1-palmitin-2-olein-3-linolein	POL	46	857.38	C_55_H_100_O_6_	2680-59-3
9	1,2-olein-3-palmitin	OOP	48	859.39	C_55_H_102_O_6_	2190-30-9
10	1,2-palmitin-3-olein	PPO	48	833.36	C_53_H_100_O_6_	1867-91-0
11	1,3-olein-2-palmitin	OPO	48	859.39	C_55_H_102_O_6_	1716-07-0
12	triolein	OOO	48	885.43	C_57_H_104_O_6_	122-32-7

ECN: equivalent carbon number, calculated as ECN = CN (number of carbon atoms) – 2DB (double bonds).

**Table 3:** Concentrations (mg/g) of TGs quantified in authenticated and commercial oils.

Sample		LLO	OLPo	LLP	PLnP	OLO	OOPo	OLP	POPo	PLP	OOO	OOP	POP	SOO	Total
		ENC 44				ENC 46					ENC 48			ENC 50	
Authenticated Sample															
Avocado	Peel	6.11	9.88	8.90	3.03	114.56	48.36	46.24	45.04	4.87	184.01	168.52	32.22	2.54	674.27
	Pulp	12.13	23.00	17.71	5.36	163.65	64.59	57.27	53.46	7.14	215.97	213.03	26.88	2.37	862.55
	Seed	ND	ND	ND	ND	ND	ND	ND	ND	ND	ND	ND	ND	ND	
Sesame		153.08	ND	53.07	ND	360.28	ND	96.16	ND	8.04	87.04	51.14	6.97	39.92	855.69
Soybean		105.65	ND	100.94	ND	130.00	ND	65.01	ND	18.16	11.16	14.07	2.10	4.57	451.67
Commercial Sample															
S1		20.08	10.19	14.80	3.52	196.21	38.00	51.55	36.95	6.57	286.11	179.71	27.78	17.39	888.87
S2		27.56	8.12	18.09	2.88	172.84	41.11	44.14	33.82	3.78	246.88	159.06	22.50	23.77	804.52
S3		13.27	8.47	11.56	4.12	137.46	38.66	40.19	39.87	6.87	265.76	179.46	29.03	22.59	797.32
S4		24.77	ND	ND	0.34	96.29	ND	ND	ND	ND	ND	20.10	3.27	18.80	163.56
S5		44.51	ND	13.41	ND	165.75	6.64	27.82	ND	1.45	377.75	97.94	4.35	51.84	791.44
S6		107.34	ND	93.88	0.58	137.12	ND	61.63	ND	14.79	14.30	13.24	1.96	6.40	451.24
S7		96.36	ND	55.88	ND	190.11	ND	53.50	ND	5.76	250.13	57.13	4.27	33.76	746.91
S8		8.66	22.47	18.53	8.29	120.27	55.97	64.96	68.08	12.79	155.42	214.73	46.91	3.49	800.57
S9		106.36	ND	104.00	0.68	134.13	ND	60.83	ND	15.26	15.09	13.43	2.05	5.30	457.14
S10		43.90	ND	8.32	ND	342.05	ND	24.27	ND	0.49	243.62	33.42	0.69	13.73	710.50
S11		47.24	ND	13.44	ND	166.03	5.03	29.59	ND	2.99	372.85	97.72	9.37	40.82	785.08
S12		9.16	20.91	20.09	8.69	139.01	54.32	53.33	68.14	11.85	204.35	213.48	35.05	4.10	842.47
S13		18.04	22.61	21.84	6.08	183.92	43.22	64.18	36.20	9.24	190.67	182.76	22.59	2.23	803.58
S14		26.41	2.20	12.33	0.67	121.38	15.54	28.78	10.28	2.16	330.82	98.37	10.09	36.80	695.84
S15		41.38	ND	8.15	ND	302.47	ND	21.06	ND	0.41	219.81	27.92	0.45	12.43	634.07
S16		97.55	ND	89.99	ND	120.85	ND	58.65	ND	13.95	9.48	11.62	1.62	4.53	408.24
S17		68.92	1.41	70.08	1.68	109.23	20.16	60.72	13.79	12.50	66.07	62.91	13.00	4.47	504.95
S18		44.27	ND	12.57	ND	145.54	5.11	23.75	0.75	1.39	364.13	82.06	4.46	43.27	727.28
S19		10.69	ND	10.13	1.41	72.82	23.30	40.16	17.31	16.65	278.46	129.25	37.57	26.84	664.59

ND: Not detected.

**Table 4:** Concentrations (mg/g) of FAs quantified in authenticated and commercial oils.

Sample		Palmitic	Palmitoleic	Stearic	Oleic	Vaccenic	Linoleic	Linolenic	Total
		* C16:0	C16:1	C18:0	C18:1 n9	C18:1 n11	C18:2 n9,12	C18:3 n9,12,15	
Authenticated Sample									
Avocado	Peel	101.76	37.26	1.79	416.79	39.82	55.09	7.24	659.76
	Pulp	138.42	67.19	1.36	538.68	52.66	89.38	5.61	893.30
	Seed	5.72	1.46	ND	11.21	1.84	14.34	3.11	37.67
Sesame		87.76	2.91	49.91	428.78	8.91	384.17	2.38	964.83
Soybean		103.50	ND	36.05	192.76	12.07	551.96	71.20	967.54
Commercial Sample									
S1		140.54	45.56	9.75	628.24	35.28	104.96	5.12	969.44
S2		135.90	44.55	17.57	600.69	32.53	122.65	4.59	958.48
S3		149.64	52.67	11.02	636.63	32.51	81.85	3.86	968.19
S4		96.24	ND	36.00	176.21	11.83	529.57	68.28	918.14
S5		57.99	2.87	18.56	780.24	10.99	124.76	2.36	997.76
S6		99.11	1.46	45.46	195.88	12.15	524.70	75.54	954.29
S7		70.61	1.70	19.69	555.30	7.45	263.92	6.65	925.31
S8		216.42	68.96	4.18	540.09	39.63	100.33	7.17	976.78
S9		103.67	1.39	37.24	189.92	12.12	539.78	73.01	957.13
S10		31.11	2.36	12.81	584.51	24.07	157.89	58.09	870.84
S11		69.93	2.80	16.78	763.75	11.01	125.40	2.46	992.14
S12		176.64	86.07	2.61	492.78	46.63	86.01	5.51	896.25
S13		140.03	60.34	1.39	472.40	54.00	126.68	7.34	862.18
S14		75.76	16.95	18.62	659.98	20.40	100.36	3.29	895.36
S15		30.87	2.45	13.00	570.62	23.34	155.03	58.48	853.80
S16		101.52	ND	38.11	177.06	11.93	523.32	77.03	928.98
S17		128.68	22.63	28.12	296.92	24.61	381.71	51.09	933.77
S18		59.78	3.24	18.18	758.07	10.49	123.22	2.86	975.85
S19		144.53	31.56	19.40	621.16	24.22	77.97	6.96	925.79

*: The formula is expressed as CN (carbon number): DB (double bond) with the position of double bond.

**Table 5:** Comparison of concentrations (weight %) of individual FA calculated from UHPLC/MS and GC/MS.

Fatty Acid	Avocado Peel		Avocado Pulp	
	LC/MS	GC/MS	LC/MS	GC/MS
Palmitic C16:0	16.31	15.42	15.35	15.59
Palmitoleic C16:1	4.81	5.65	5.13	7.57
Stearic C18:0	0.36	0.27	0.21	0.15
Oleic & Vaccenic C18:1	68.15	69.21	66.98	66.21
Linoleic C18:2	10.21	8.35	12.11	10.07
Linolenic C18:3	0.16	1.10	0.22	0.63

## Supplementary Materials

The following are available online: Table S1: Information of commercial avocado (*P. americana*) oils.
