# Supplementary material for: Characterization, Quantification and Quality Assessment of Avocado (Persea americana Mill.) Oils
Source: Molecules. 2020 Mar 24;25(6):1453. doi: 10.3390/molecules25061453 (PMC7145317; doi:10.3390/molecules25061453)
Supplement: Supplementary file 1 [file molecules-25-01453-s001.zip › molecules-733494-SI.docx]

**SUPPLEMENTAL MATERIAL**

Characterization, Quantification and Quality Assessment of Avocado (*Persea americana* Mill.) Oils

Mei Wang^1^, Ping Yu^2,3,4^, Amar G. Chittiboyina^1^, Dilu Chen^5^, Jianping Zhao^1^, Bharathi Avula^1^, Yan-Hong Wang^1^, Ikhlas A. Khan ^1,6^

Affiliations

1National Center for Natural Products Research, School of Pharmacy, University of Mississippi, University, MS 38677, USA

2State Key Laboratory of Food Science and Technology, Nanchang University, Nanchang 330031, China

3Jiangxi Province Key Laboratory of Edible and Medicinal Resources Exploitation, Nanchang University, Nanchang 330031, China

4School of Resource and Environmental and Chemical Engineering, Nanchang University, Nanchang 330031, China

5School of Pharmacy, Hunan University of Chinese Medicine, Changsha 410208, China

6Division of Pharmacognosy, Department of BioMolecular Sciences, School of Pharmacy, University of Mississippi, University, MS 38677, USA

Correspondence

Dr. Ikhlas A. Khan

Phone: + 16629157821, Fax: + 16629157062

ikhan@olemiss.edu

**Table S1**. Information of commercial avocado (*P. americana*) oils

| **No.** | **Product**  **Name** | **Extracted from Plant Part** | **Label Claim** |
| --- | --- | --- | --- |
| S1 | Avocado oil | Pulp | All natural and expeller-pressed from the pulp of avocados. Contain high level of monosaturated fats. Use for cooking , moisturizing skin and hair |
| S2 | Avocado oil | Not specified | Use as a natural face and body moisturizer or for massage. Can also be used as a hair conditioner |
| S3 | Avocado oil | Not specified | Cold pressed refined avocado oil, for roasting, dressing and sautéing |
| S4 | Avocado seed oil | Seed | Extract to grow hair, ingredients includes extract mineral oil, avocado seed oil, fragrance, color and preservatives |
| S5 | Avocado oil | Not specified | 100% pure and organic cold pressed avocado oil. Cruelty free and preservative free. For all skin and hair types |
| S6 | Avocado oil | Not specified | 100% pure and organic cold pressed avocado oil. Use for hair, skin and suitable for food and cosmetics |
| S7 | Avocado oil | Not specified | No claim |
| S8 | Avocado oil | Not specified | 100% pure moisturizing avocado oil. Use as a scalp or skin conditioner, or as an emollient. For soft, healthy skin, nutrient rich and hydrating |
| S9 | Avocado seed oil | Seed | No claim |
| S10 | Avocado oil | Seed | 100% pure and organic, cold pressed seed avocado oil, food grade, is repackaged for cosmetic use |
| S11 | Avocado oil | Not specified | Extracted from fresh avocado fruit, 100% pure, for healthy hair, skin and nail |
| S12 | Avocado oil | Not specified | 100% natural oil, for external use only |
| S13 | Avocado oil | Not specified | Extra virgin, California grown avocados, ideal for baking, sautéing, grilling, dipping or as an ingredient in homemade salad dressing |
| S14 | Avocado oil | Not specified | Naturally refined, high-heat cooking oil (500 °F), NSF certified |
| S15 | Avocado oil | Not specified | 100% pure, cold pressed, refined, organic and grade A avocado oil |
| S16 | Avocado oil | Not specified | 100% pure, cold pressed, refined, organic and grade A avocado oil |
| S17 | Avocado oil | Pulp | Pure and natural, cold pressed and unrefined avocado oil from fruit pulp |
| S18 | Avocado oil | Not specified | Natural pure organic therapeutic avocado oil, strengthen skin and assist scalp and hair growth |
| S19 | Avocado seed oil | Seed | 100% pure cold pressed oil, use for dry skin, a general body oil or a nourishing hair and scalp massage |
